# Leptospirosis in the Delta Region of Tamil Nadu: A Retrospective Analysis in South India

**DOI:** 10.7759/cureus.98096

**Published:** 2025-11-29

**Authors:** P B Praveen Kumar, Eunice Swarna Jacob, A Shanthi

**Affiliations:** 1 Department of Microbiology, Thanjavur Medical College, Thanjavur, IND

**Keywords:** infectious causes of febrile jaundice apart viral hepatitis, leptospirosis with severe clinical manifestation, rodent control, south india population, tamil nadu population

## Abstract

Leptospirosis is a bacterial infection that targets multiple systems in our human body, and we are witnessing this in our routine clinical practice and ward rounds. In Thanjavur, which is commonly referred to as the Delta region of Tamil Nadu, agricultural workers, sewage workers, and all those individuals who are liable to work in rodent-infected environments are at increased risk. We found the prevalence of leptospirosis to be 17% (n = 85) during our study period from March 2024 to December 2024. Even though the prevalence was found to be considerably low, preventive measures like proper waste disposal, rodent control, and health education should be regularly taken at all levels.

## Introduction

Leptospirosis is a syndrome of severe multisystem disease presenting with severe jaundice and kidney function impairment. Historically, Leptospira was classified into two species: *Leptospira interrogans* and *Leptospira biflexa*. According to the DNA-DNA hybridization (DDH) and modern 16S ribosomal RNA phylogenetic classification, the genus Leptospira is divided into three clades, namely, pathogen, intermediate, and saprophytic clades. Pathogenic Leptospira (*Leptospira interrogans*) falls in subgroup I of the pathogenic clade. Recently, modern genomic studies, overtaking DDH classification of *Leptospira* and reclassification, have been proposed, which contain two major clades: Pathogenic (P) and Saprophytic (S). Future studies would throw more insight into this [1].

Leptospirosis is endemic to the Indian subcontinent, especially in southern states [2]. Leptospirosis is one of the zoonotic infections, and it is one of 17 neglected tropical infections provided by the World Health Organization [3,4].

In our study, seroprevalence of leptospirosis was found to be 17% (n = 85). In India, the high burden of leptospirosis has been reported from more than five states, including Tamil Nadu, and outbreaks are more common following the monsoon rainy season [1].

## Materials and methods

Study design and place

This was a retrospective cross-sectional study carried out from March 2024 to December 2024 at Thanjavur Medical College Hospital, India.

Inclusion criteria

This analysis included patients of all age groups from various outpatient departments and wards who presented with clinical signs and symptoms such as fever, vomiting, abdominal pain, yellowish discoloration of the eyes, and loss of appetite.

Exclusion criteria

Patients with confirmed (both laboratory and clinical) cases of other fever causes like dengue, chikungunya, scrub typhus, and typhoid, including paratyphoid fever, were likely excluded.

Sample collection

Patients who presented with fever and demographic characteristics, including area of residence, socioeconomic status, and source of drinking water, were elicited and documented in the case record form. Under strict aseptic precautions, 5 mL of venous blood was collected in a sterile vacutainer after obtaining informed consent from the patients for the assessment of IgM antibodies against leptospirosis.

Sample rejection criteria

Following appropriate discussion with the concerned medical team, samples that were collected in wrong or irrelevant containers and those that were not labeled properly were rejected.

Sample processing

Blood samples were routinely received, centrifuged for 20 minutes at 2,000 revolutions per minute (rpm), and the serum was aliquoted into individual vials and labeled appropriately. As directed in the kit insert, IgM was detected using a commercially available enzyme-linked immunosorbent assay (ELISA) test kit (Abbott, Gyeonggi-do, Republic of Korea). Every test run was carried out with the appropriate calibrators. The optical density was measured using a calibrated ELISA reader. The validity of the test runs and the results was reviewed in accordance with the manufacturer's instructions. A negative result means that the patient is not experiencing an acute leptospirosis infection and that there are no detectable IgM antibodies against Leptospira in the sample. One week following the initial examination, a second sample should be examined for any patient exhibiting an equivocal result. A leptospirosis infection is indicated by a positive test; thus, the patient should receive the appropriate care. The findings were recorded and statistically examined.

Data analysis

Descriptive statistics like frequency counts and percentages were used to describe and assess the results. The laboratory for microbiology processed the samples that were taken from patients who had leptospirosis-like symptoms, including those in wards and outpatient departments. Patient information, including age, gender, place of residence, and diagnosis, was recorded using the requisition form that was obtained by the microbiological lab. We gathered additional patient-related information from the patient case sheet, hospital medical records, and phone calls, such as symptoms and socioeconomic status. Spreadsheets from Microsoft Excel (Microsoft Corporation, Redmond, Washington) were used to report and reorganize the data gathered from each patient. Summary tables were used to summarize the number of positive instances in each group after the patients were divided into other groups, such as age and gender, for descriptive analysis.

Ethical considerations

Since this is a retrospective study, broader consent and assent were obtained from patients and patient caregivers, respectively, at the time of data collection through telephone conversations, indicating potential future research use of their data.

## Results

**Figure 1:**
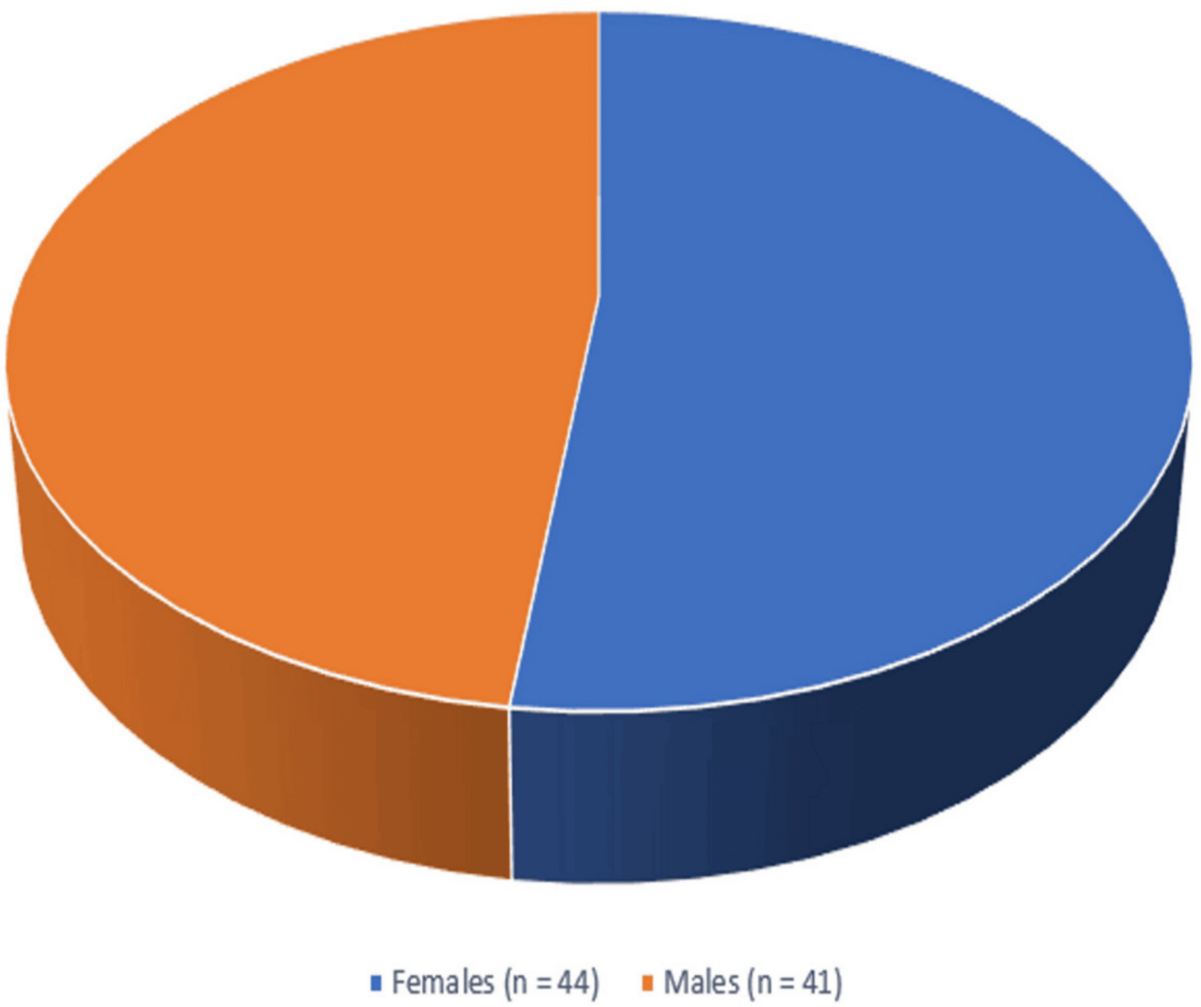
Gender-wise distribution of leptospirosis-positive cases

**Figure 2:**
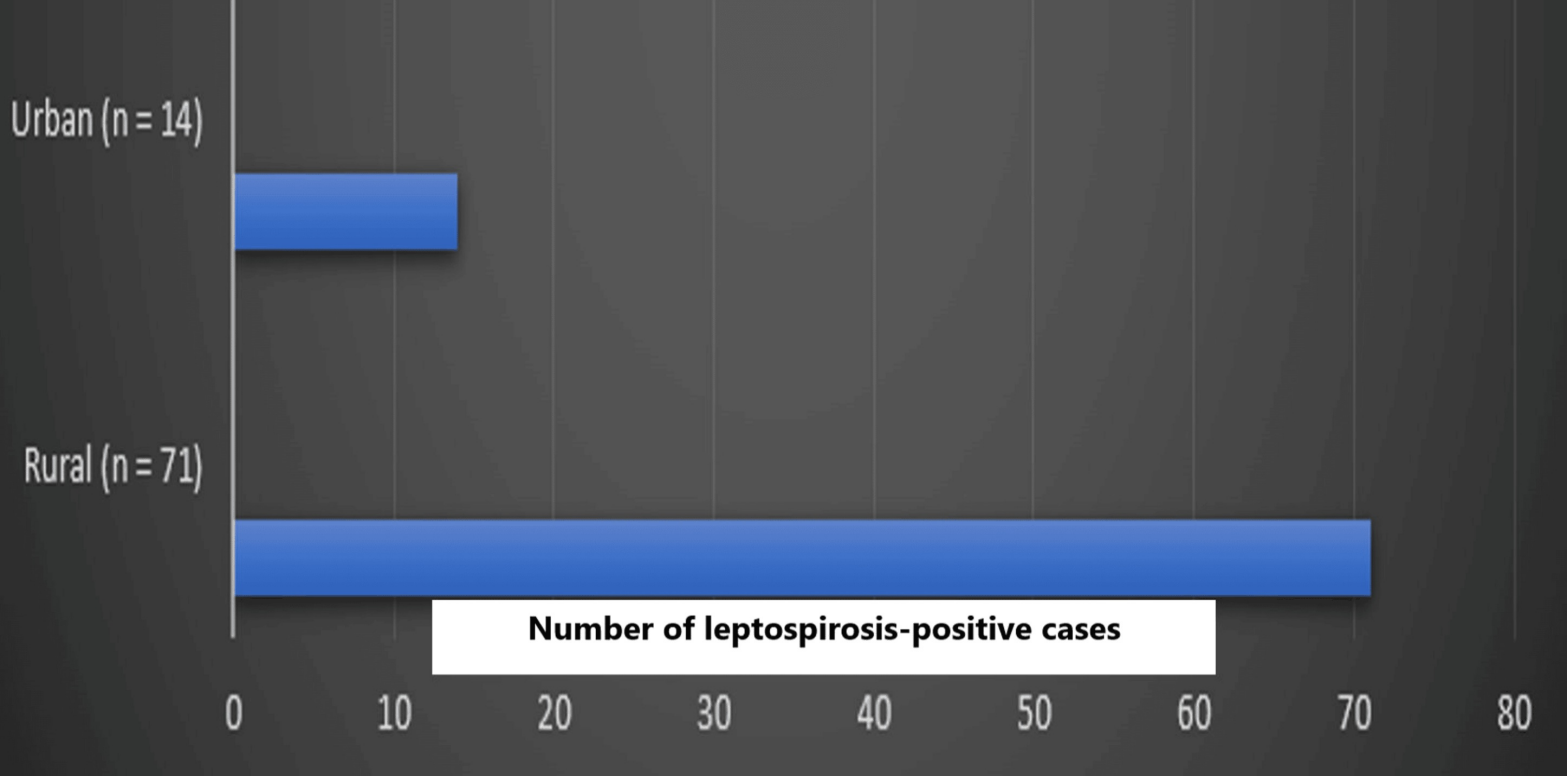
Residential-wise distribution of leptospirosis-positive patients

A total of 500 serum samples were received for analysis. Out of 500 samples tested, 17% (n = 85) tested positive for IgM antibodies against leptospirosis. Figure 1 shows that there is a slight preponderance of positive cases in female patients (52%, n = 44) compared to male patients (48%, n = 41). Age-wise distribution shows that the 21-30-year-old age group tops the list with a high number of positive cases (38%, n = 32), followed by 11-20 years (25%, n = 21), ≤10 years (21%, n = 18), 31-40 years (8%, n = 7), and ≥40 years (8%, n = 7), respectively (Table 1). Among 85 leptospirosis-positive cases, 71 cases were residing in rural areas, and 14 cases were residing in urban areas (Figure 2). Most of the patients presented with fever, abdominal pain, and jaundice. About 80% (n = 68) of the leptospirosis-positive cases were using tap water for drinking. All positive cases were treated with doxycycline and cephalosporins, which led to a favorable outcome. Since all positive cases reported in this study were reported at an early stage of their illness, it paved the way for earlier and positive outcomes.

**Table 1:** Age-wise distribution of leptospirosis-positive cases ELISA: enzyme-linked immunosorbent assay; IgM: immunoglobulin M

Age category (in years)	Leptospirosis IgM ELISA-positive cases (n = 85)	Percentage of people with leptospirosis
≤10	18/85	21
11-20	21/85	25
21-30	32/85	38
31-40	7/85	8
>40	7/85	8

## Discussion

Leptospirosis is a global disease, with an estimated yearly incidence of over one million cases and nearly 60,000 fatalities. The incidence of leptospirosis is much higher in warm climate countries than in temperate regions [1]. In our study, we detected a prevalence of leptospirosis of 17% (n = 85). In a study done at Villupuram, where fever cases reported in private clinics were included in the study, 65 positive cases (4.3%) were detected from 1,502 samples by means of both the macroscopic slide agglutination test (MSAT) and IgM ELISA [5]. Similarly, a study conducted by the Tamil Nadu Dr. MGR Medical University, Chennai, Guindy, India, reported around 19.5% (n = 748) out of 3,830 serum samples received from different locations in and around Chennai. But in that study, they utilized the microscopic agglutination test (MAT) and dark field microscopy [6]. In another study done at a place that is just 60 km away from our study location, 66% of the suspected cases were confirmed as leptospirosis by both MAT and IgM ELISA [7].

Natarajaseenivasan et al. conducted a study among rice mill workers in Salem, and it is suggested that they had a seroprevalence rate of around 68.3%, which is higher than that of persons engaged in other groups [8]. A six-year retrospective study conducted at Theni, near Madurai, a famous tourist spot in Tamil Nadu, showed that only 3.2% of cases were diagnosed with leptospirosis using IgM ELISA [9]. In Erode, 29 suspected leptospirosis cases were subjected to culture and serological evaluation, and the results show 24.1% positivity and 89.7% positivity in culture and serological evaluation, respectively [10]. In a village near the Kolli Hills, Namakkal, out of 540 people in the area, only three tested positive for leptospirosis due to water from an unprotected well [11]. In another study conducted in Chennai, 100 samples were collected from patients with acute febrile illness. Among 100 samples, 21% (n = 21) tested positive for leptospirosis [12]. Compared with our study, all previously mentioned research fell roughly above or below the leptospirosis rate in our hospital.

For accurate diagnosis, all three parameters, including clinical, epidemiological, bacteriological, and laboratory findings, were analyzed based on Modified Faine’s criteria (Table 2) [13]. The limitations of standard serological testing make early detection difficult, highlighting the necessity of increasing access to polymerase chain reaction-based diagnostics in endemic areas. Better surveillance, prompt supportive care, and risk-based intensive care unit resource allocation are critical, given the noted geographical diversity in illness severity [14]. Depending on the severity of leptospirosis, treatment regimens may vary from doxycycline to cell-wall-acting agents like ampicillin, penicillin, and cephalosporins. For patients who are showing an allergy to the penicillin group of drugs, azithromycin may be indicated [1].

**Table 2:** Modified Faine's criteria A presumptive diagnosis of leptospirosis is made when either of the criteria is met Part A or Part A and Part B score: ≥26 Parts A, B, and C (total): ≥25 A possible diagnosis of leptospirosis is made when a score is between 20 and 25 [13] PCR: polymerase chain reaction; ELISA: enzyme-linked immunosorbent assay; IgM: immunoglobulin M; MSAT: macroscopic slide agglutination test; MAT: microscopic agglutination test

Criteria	Scoring
Part A: clinical criteria	
Headache	2
Fever	2
Fever >39°C	2
Conjunctival suffusion	4
Meningism	4
Myalgia	4
Conjunctival suffusion + meningism + myalgia	10
Jaundice	1
Albuminuria/nitrogen retention	2
Haemoptysis/dyspnea	2
Part B: epidemiological factors	
Rainfall	5
Contact with contaminated environment	4
Animal contact	1
Part C: bacteriological and laboratory findings	
Isolation of Leptospira in culture or detection in PCR	25
Positive serology	
ELISA IgM positive	15
MSAT positive	15
Other rapid tests	15
MAT: single positive in high titer	15
MAT: rising titer/seroconversion (paired sera)	25

Limitations and strengths

One of the main limitations of the study is that, due to the retrospective design, we are unable to draw conclusions about risk factors and their association with leptospirosis. Additionally, only one serological test was used for all samples in this study. We did not test and confirm leptospirosis using MSAT, MAT, and culture. However, to the best of our knowledge, this is the first study to estimate the prevalence of leptospirosis in our locality.

## Conclusions

Leptospirosis can be prevented by developing trained manpower for diagnosis, case management, and intersectoral coordination. Strengthening the surveillance and diagnostic capacity of laboratories plays an important role in endemic areas. Creating awareness among the local public regarding timely detection and appropriate treatment of patients also plays a major role in the control of the disease. Antirodent measures need to be actively implemented, like eliminating nesting areas and sealing the entry points of rodents, rodenticide utility and ultrasonic rodent repellents, and improved hygienic practices.
